# The effects of 6-gingerol on proliferation, differentiation, and maturation of osteoblast-like MG-63 cells

**DOI:** 10.1590/1414-431X20154494

**Published:** 2015-04-28

**Authors:** J.Z. Fan, X. Yang, Z.G. Bi

**Affiliations:** Department of Orthopedic Surgery, First Affiliated Hospital, Harbin Medicine University, Harbin, China

**Keywords:** 6-Gingerol, Interleukin-6, Nuclear factor-κB, Osteogenesis, Osteoblast

## Abstract

We investigated whether 6-gingerol affects the maturation and proliferation of osteoblast-like MG63 cells *in vitro*. Osteoblast-like MG63 cells were treated with 6-gingerol under control conditions, and experimental inflammation was induced by tumor necrosis factor-α (TNF-α). Expression of different osteogenic markers and cytokines was analyzed by real-time PCR, Western blotting, and enzyme-linked immunosorbent assay. In addition, alkaline phosphatase (ALP) enzyme activity and biomineralization as markers for differentiation were measured. Treatment with 6-gingerol resulted in insignificant effects on the proliferation rate. 6-Gingerol induced the differentiation of osteoblast-like cells with increased transcription levels of osteogenic markers, upregulated ALP enzyme activity, and enhanced mineralized nodule formation. Stimulation with TNF-α led to enhanced interleukin-6 and nuclear factor-κB expression and downregulated markers of osteoblastic differentiation. 6-Gingerol reduced the degree of inflammation in TNF-α-treated MG-63 cells. In conclusion, 6-gingerol stimulated osteoblast differentiation in normal physiological and inflammatory settings, and therefore, 6-gingerol represents a promising agent for treating osteoporosis or bone inflammation.

## Introduction

Osteoporosis is a metabolic bone disorder characterized by deteriorated bone tissue and low bone mass, leading to an increased risk of fracture (1). It is prevalent in the elderly population, particularly women, with an increasing incidence worldwide (2). The disorder is caused by an imbalance in bone resorption *vs* formation due to aging and insufficient estrogen production. Osteoporosis is treated with the goal of reducing fracture risk by inhibiting bone resorption or stimulating bone formation. However, current treatments for osteoporosis are limited to drugs that act predominantly to inhibit bone resorption, such as bisphosphonates. Despite the documented clinical benefits, anti-resorptive agents have been reported to have unwanted side effects, including gastrointestinal adverse effects, breast cancer, and deep venous thromboembolic disease (3-5). In contrast, bone anabolic therapy stimulating bone formation via actions on osteoblasts represents an ideal therapeutic approach for osteoporosis (6-8). In addition to synthetic agents, there has been increasing focus by researchers on alternative therapies such as phytomedicines (9). There is, thus, a great need to scientifically evaluate food ingredients that stimulate bone formation as preventive or therapeutic agents for osteoporosis.

Ginger, the rhizome of *Zingiber officinale*, has a long history of use as a traditional medicine in many countries. Its extracts have been used for managing a variety of ailments, including inflammation, infection, constipation, indigestion, and hypertension (10-13). Several bioactive ingredients have been identified in ginger, including gingerols and gingerol-related compounds (14). Among polyphenolic compounds, zerumbone, a gingerol-related compound, has been shown to inhibit osteoclastogenesis induced by receptor activator of nuclear factor-κB (NF-κB) ligand (RANKL) (15). Recent research has reported that 10-gingerol induces the bone morphogenetic protein signaling pathway in a model of zebra fish (16). Ginger has been demonstrated to be effective against bone-related disorders such as osteoarthritis (17,18). Taken together, it is suggested that ginger theoretically has the potential for beneficial effects on bone metabolism; however, its effects on bone metabolism are sketchy.

In the present study, we investigated the effect of 6-gingerol, a predominant bioactive ingredient of ginger, on bone metabolism with an emphasis on bone formation. We determined the effect of 6-gingerol on proliferation and differentiation of osteoblasts by measuring alkaline phosphatase (ALP) activity, collagen type I synthesis, and bone mineralization in 6-gingerol-treated human osteoblast-like MG-63 cells. We also studied the osteoprotective effects of 6-gingerol on tumor necrosis factor-α (TNF-α)-treated osteoblasts.

## Material and Methods

### Cell culture

Human osteoblast-like MG63 cells (ATCC CRL-1427) were obtained from American Type Culture Collection (USA) and maintained in Dulbecco's modified Eagle's medium (DMEM; Gibco BRL, USA) supplemented with 10% fetal bovine serum (FBS; Gibco BRL) and 100 µg/mL penicillin-streptomycin at 37°C in a humidified atmosphere containing 5% CO_2_. Cells were seeded on six-well culture plates at an initial density of 1×10^5^ cells/mL and grown to approximately 80% confluence. For treatment with 6-gingerol, MG-63 cells were pretreated with 1% FBS DMEM for 16 h, and then treated with TNF-α (10 ng/mL) followed by exposure to 6-gingerol (Sigma, USA) with serial concentrations (0-50 µM) in serum-free DMEM for 24 or 48 h. After the 6-gingerol treatment, the cells were washed with phosphate-buffered saline (PBS; 25 mM sodium phosphate, 150 mM NaCl, pH 7.2) and collected for the following analyses.

### ALP activity assay

ALP activity was measured by analyzing the rate of p-nitrophenyl phosphate (Sigma) hydrolysis. Cells were seeded at a density of 1×10^5^cells/well on 24-well plates and incubated overnight. The cells were treated with 6-gingerol at various concentrations in DMEM and incubated for 72 h. The medium was removed, and 500 µL of 0.1% Triton X-100 (Sigma) was added to each well. The cells were frozen at −70°C and defrosted at 37°C, followed by centrifugation at 14,000 *g* at 4°C for 10 min. The supernatant was collected for measuring ALP activity. The absorbance at 405 nm was measured using a microplate reader (FlexStation3, Molecular Devices, USA). Protein levels were quantified using the bicinchoninic acid assay.

### RNA extraction and real-time PCR

Total RNA was extracted from cell cultures using Trizol (Sigma) according to the manufacturer's protocol. The mRNA levels of the genes analyzed were measured by real-time PCR amplification. Sequences for mRNAs from the nucleotide data bank (National Center for Biotechnology Information, USA) were used to design primer pairs for real-time PCR reactions (Primer Express, Applied Biosystems, USA). The specific oligonucleotide primers used in this study are listed in Table 1. Real-time PCR cycles were adjusted to have linear amplification for all the targets. Each real-time PCR reaction was repeated at least three times.

**Table:**
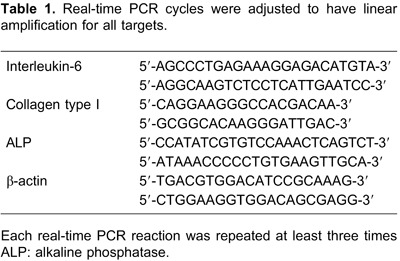

### Western blot analysis

Cells were scraped off into culture medium and collected by centrifugation at 4000 *g* for 15 min before resuspension in the lysis buffer. Cell lysate was subjected to a 12% SDS-PAGE gel and subsequently transferred onto a nitrocellulose membrane as previously described. The resulting blot was incubated in 5% nonfat milk in PBS for 1 h and then probed with a primary antibody against collagen type I and NF-κB p65, followed by reacting with an appropriate peroxidase-conjugated secondary antibody for 1 h. Signals were detected using an enhanced chemiluminescence detection kit (Amersham Biosciences, USA) and were quantitated by an image analysis system (Fuji Film, Japan).

### Subcellular fractionation

Cells were washed with PBS and incubated with a lysis buffer (10 mM HEPES, pH 7.6) for 10 min. Cell lysates were centrifuged at 2,500 *g* for 10 min at 4°C. The supernatant containing the cytosol, i.e., the cytosolic fraction, was further centrifuged at 20,000 *g* for 15 min at 4°C. The pellets containing nuclei were washed with PBS, resuspended in nuclear buffer (25 mM HEPES, pH 7.6), and centrifuged at 10,000 *g* for 15 min at 4°C. The resulting supernatant, i.e., the nuclear fraction, was collected.

### Enzyme-linked immunosorbent assay (ELISA)

Concentrations of collagen type I and interleukin (IL)-6 in supernatants of osteoblast cultures were measured by ELISA according to the manufacturer's instructions. The level of IL-6 in the culture medium was detected using a human IL-6 Quantikine ELISA Kit (R&D Systems, USA). The concentration of collagen type I was measured using type I collagen using a Procollagen-C ELISA kit (Metra Biosystems, USA).

### Statistical analysis

Data are reported as means±SD of three independent experiments. Statistical comparisons were made by Student's *t*-test or one-way analysis of variance, followed by Duncan's multiple-comparison test. Differences were considered to be significant when P<0.05.

## Results

### Effect of 6-gingerol on viability of osteoblast-like MG-63 cells

**Figure 1:**
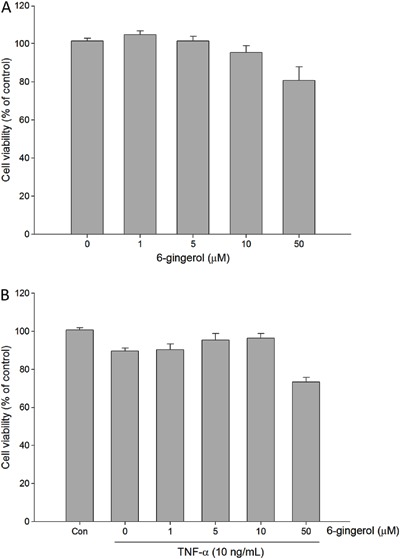
Effects of 6-gingerol on cell viability of osteoblast-like MG6-3 cells. *A*, MG-63 cells were treated with serial concentrations of 6-gingerol (0-50 µM) for 48 h. *B*, MG-63 cells were stimulated with tumor necrosis factor (TNF)-α (10 ng/mL) and treated with serial concentrations of 6-gingerol (0-50 µM) for 48 h. Cells were measured for viability. Data are reported as means±SE for 3 independent experiments. There were no significant differences compared to control (Con) (Student's *t*-test).

To determine whether 6-gingerol causes cytotoxicity in osteoblasts, osteoblast-like MG-63 cells were incubated with serial concentrations of 6-gingerol (0-50 μM) for 48 h, and cell viability was determined using an MTT assay. No significant change in cell viability was observed in the presence of 6-gingerol at tested concentrations (Figure 1A). We further evaluated the cytotoxic effect of 6-gingerol on TNF-α-treated MG-63 cells. Treatment of MG-63 cells with TNF-α (10 ng/mL) resulted in a slight decrease in cell viability, and 6-gingerol exerted no significant influence on the viability of TNF-α-treated MG-63 cells (Figure 1B).

### Effect of 6-gingerol on MG-63 differentiation

**Figure 2:**
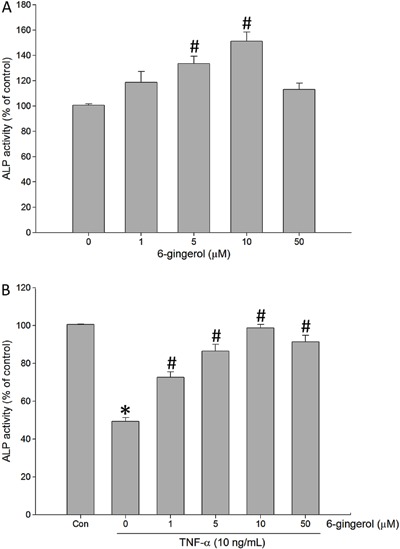
6-Gingerol stimulated alkaline phosphatase (ALP) activity in MG-63 cells treated without (*A*) or with (*B*) stimulation of tumor necrosis factor (TNF)-α. Cells were treated with serial concentrations of 6-gingerol (0-50 µM) for 48 h. Data are reported as means±SE for 3 independent experiments. ^#^P<0.05 compared to 0 µM; *P<0.05 compared to control (Con) (Student's *t*-test).

**Figure 3:**
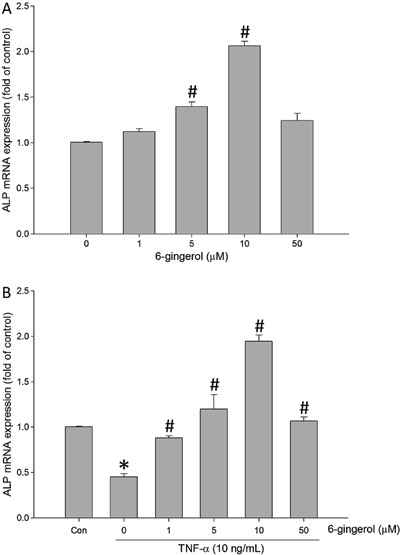
6-Gingerol upregulated alkaline phosphatase (ALP) mRNA expression in MG-63 cells treated without (*A*) or with (*B*) stimulation of tumor necrosis factor (TNF)-α. Cells were treated with serial concentrations of 6-gingerol (0-50 µM) for 48 h and mRNA expression was determined by real-time PCR. Data are reported as means±SE for 3 independent experiments. ^#^P<0.05 compared to 0 µM; *P<0.05 compared to control (Con) (Student's *t*-test).

We investigated the effects of 6-gingerol on osteoblast differentiation by measuring the expression and enzyme activity of ALP, which is involved in the maturation step of osteoblasts. As shown in Figure 2A, treatment with 6-gingerol increased enzyme activity of ALP in MG-63 cells in a dose-dependent manner. ALP activity was significantly reduced in response to TNF-α stimulation (10 ng/mL), and the decreased activity of ALP was elevated by 6-gingerol dose-dependently (Figure 2B). Quantitative analysis using real-time PCR revealed that 6-gingerol upregulated mRNA expression of ALP in MG-63 and restored the reduction of ALP expression in TNF-α-stimulated MG-63 cells (Figure 3).

**Figure 4:**
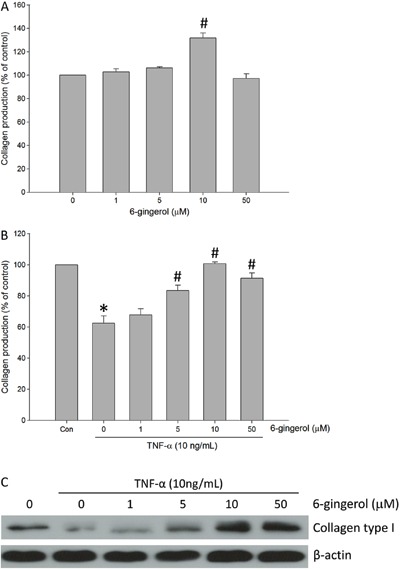
6-Gingerol stimulated collagen synthesis in MG-63 cells treated without (*A*) or with (*B*) stimulation of tumor necrosis factor (TNF)-α. Cells were treated with serial concentrations of 6-gingerol (0-50 µM) for 48 h and collagen synthesis in MG-63 cells was determined by ELISA. *C*, Intracellular collagen production was determined using immunoblotting. Data are reported as means±SE for 3 independent experiments. ^#^P<0.05 compared to 0 µM; *P<0.05 compared to control (Con) (Student's *t*-test).

**Figure 5:**
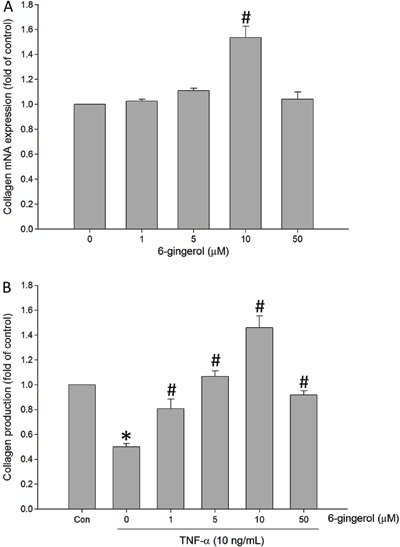
6-Gingerol upragulated collagen type I mRNA expression in MG-63 cells treated without (*A*) or with (*B*) stimulation of tumor necrosis factor (TNF)-α. Cells were treated with serial concentrations of 6-gingerol (0-50 µM) for 48 h and mRNA expression was determined by real-time PCR. Data are reported as means±SE for 3 independent experiments. ^#^P<0.05 compared to 0 µM; *P<0.05 compared to control (Con) (Student's *t*-test).

We next determined the effects of 6-gingerol on the synthesis of collagen type I, which is considered an essential event in early osteoblast differentiation. Synthesis of collagen type I in MG-63 cells was increased by treatment with 6-gingerol at a concentration of 10 µM, whereas 50 µM of 6-gingerol had no influence on collagen type I synthesis (Figure 4A). TNF-α treatment (10 ng/mL) significantly inhibited production of collagen type I in MG-63 cells (P<0.05; Figure 4B). Treatment of TNF-α-stimulated MG-63 cells with 6-gingerol resulted in a significant increase in the production of collagen type I in a dose-dependent manner (Figure 4B). The intracellular level of collagen type I was measured using immunoblotting (Figure 4C). In parallel, the level of upregulated expression of collagen in mRNA was observed in MG-63 cells treated with 6-gingerol at a concentration of 10 µM (Figure 5A). Restoration of reduced collagen mRNA expression in TNF-α-stimulated MG-63 cells shared the same pattern with that of protein production (Figure 5B).

### Effect of 6-gingerol on IL-6 production and regulation of NF-κB in MG-63 cells

**Figure 6:**
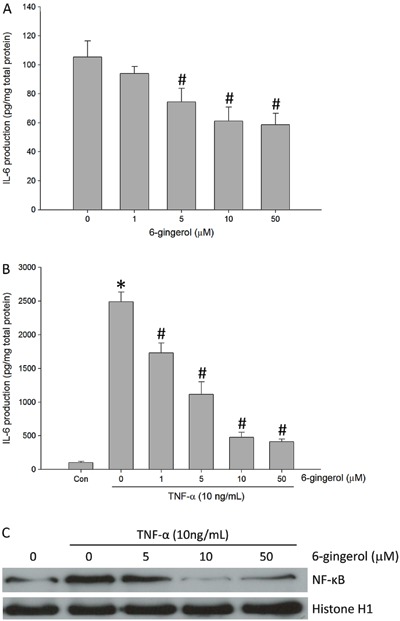
6-Gingerol suppressed interleukin (IL)-6 in MG-63 cells treated without (*A*) or with (*B*) tumor necrosis factor (TNF)-α stimulation. Cells were treated with serial concentrations of 6-gingerol (0-50 µM) for 48 h and IL-6 production was determined by ELISA. *C*, Nuclear translocation of nuclear factor (NF)-κB upon TNF-α stimulation was examined using immunoblotting. Data are reported as means±SE for 3 independent experiments. ^#^P<0.05 compared to 0 µM; *P<0.05 compared to control (Con) (Student's *t*-test).

We investigated whether 6-gingerol has a modulatory effect on IL-6 production in osteoblasts, which is reported to have a significant impact on osteogenesis. A low level of IL-6 was detected in the culture medium of untreated MG-63 cells. Treatment with 6-gingerol resulted in a significant decrease in the baseline IL-6 level (P<0.05; Figure 6A). TNF-α induced 25-fold increases in the level of IL-6 in MG-63 cells (Figure 6B). The results showed that 6-gingerol significantly suppressed the elevation of IL-6 induced by TNF-α in MG-63 cells. We next investigated the influence of 6-gingerol on the nuclear translocation of NF-κB in response to TNF-α stimulation. Immunoblotting analyses showed that TNF-α induced a significant increase in the nuclear translocation of NF-κB p65 in MG-63 cells (Figure 6C). Increases in NF-κB p65 nuclear translocation were inhibited by 6-gingerol (5, 10, and 50 µM; Figure 6C).

## Discussion

In the present study, we investigated the stimulatory effects of 6-gingerol on the proliferation and differentiation of human osteoblast-like MG-63 cells. The results showed that 6-gingerol exerted no cytotoxic effects on MG-63 cells while incubating at concentrations up to 50 μM. 6-Gingerol induced cell differentiation, with evidence of increased ALP activity and elevated collagen synthesis in MG-63 cells. Our data revealed that 6-gingerol exerted anti-inflammatory activity in MG-63 cells stimulated with TNF-α in association with the aforementioned effect on osteogenesis.

Osteoblast differentiation is characterized by three stages, including proliferation, matrix maturation, and matrix mineralization (19). ALP is one of the major enzymes expressed during the early maturation of osteoblasts, and ALP activity is considered a marker for the early differentiation of osteoblast cells (20). An increase in ALP activity was observed in osteoblast-like MG-63 cells after 6-gingerol treatment for 48 h, indicating that 6-gingerol induced the differentiation of early-stage osteoblasts. It is known that osteoblasts synthesize various proteins to make bone, including collagen, which is the predominant protein in bone matrix (21). Increases in collagen content reflect the maturation process of osteoblasts. Collagen synthesis in MG-63 cells was markedly increased in response to treatment with 6-gingerol for 48 h, suggesting that 6-gingerol enhances osteoblastic maturation. The present findings revealed the stimulatory effects of 6-gingerol on differentiation in MG-63 cells in terms of cell viability, ALP activity, and collagenogenesis, suggesting that 6-gingerol could induce differentiation in osteoblasts.

TNF-α is a proinflammatory cytokine that contributes to bone loss in conditions such as inflammatory osteolysis, periodontal inflammation, rheumatoid arthritis, and postmenopausal osteoporosis (22-24). There are two mechanisms by which TNF-α acts on the homeostasis of bone. TNF-α contributes to bone resorption by promoting osteoclast differentiation and activation through induction of RANKL, macrophage colony-stimulating factor (M-CSF), and IL-1 (25,26). Additionally, TNF-α exerts anti-apoptotic activity on osteoclasts, leading to a prolonged life span of osteoclasts (27). TNF-α also has an inhibitory influence on bone formation through the induction of osteoblast apoptosis and the inhibition of osteoblast differentiation (28-30). In the present study, a slight reduction in cell viability in MG-63 cells treated with TNF-α was observed, which was restored by 6-gingerol treatment. 6-Gingerol has been reported to exert potent anti-apoptotic activity *in vitro* and *in vivo*. It is suggested that 6-gingerol restores TNF-α-suppressed cell viability through its anti-apoptotic effect. Despite the lower influence on viability, TNF-α treatment led to a significant reduction in ALP activity and collagen synthesis in MG-63 cells. The reduction was ameliorated in response to 6-gingerol treatment, suggesting that 6-gingerol could protect osteoblasts from TNF-α-suppressed differentiation.

IL-6, a member of the gp130 cytokine family, is recognized as a potent stimulator of osteoclast-mediated bone absorption (31,32). Osteoblasts are the predominant source of IL-6 that acts on osteoclasts and bone resorption (33). It has been reported that excessive IL-6 leads to impaired microarchitecture of cortical and trabecular bone in association with increased osteoclastogenesis and decreased osteoblastogenesis (34). In clinical practice, upregulated expression of IL-6 has been reported in the bone samples of postmenopausal women with osteoporosis (35). Postmenopausal women with osteoporosis exhibit high levels of IL-6 in the serum (36). A mechanism of action of IL-6 on bone homeostasis has been suggested, in which IL-6 binds to its receptor and stimulates osteoblastic downstream expression of RANKL, which subsequently enhances osteoclast formation and activity (37). Moreover, the activity of IL-6 on osteoclasts frequently interplays with IL-1 and TNF, leading to bone resorption by increasing the osteoclastic progenitor pool (38). In this study, exposure of MG-63 to TNF-α resulted in a significantly increased level of IL-6 and a marked nuclear translocation of NF-κB. The elevations were reduced in response to 6-gingerol treatment. Because 6-gingerol has been reported to have anti-inflammatory properties, the reduction in TNF-α-stimulated IL-6 production and NF-κB nuclear translocation is putatively attributed to the immunomodulatory activity of 6-gingerol.

In conclusion, the present results demonstrated that 6-gingerol stimulated the differentiation of osteoblast-like MG-63 cells. 6-Gingerol ameliorated TNF-α-suppressed osteoblast differentiation. It is suggested that 6-gingerol may have beneficial effects on bone formation as a therapeutic agent for treating bone disorders. The anti-inflammatory activity adds value to the use of 6-gingerol as an orthopedic therapy. Further studies are needed to elucidate the underlying mechanism by which 6-gingerol stimulates osteoblastic differentiation.
